# Associations between multidimensional fatigue and wearable-derived cardio-respiratory variables in post-COVID-19 patients: an observational study

**DOI:** 10.3389/fdgth.2026.1804993

**Published:** 2026-07-16

**Authors:** Neusa R. Adão Martins, Simon Annaheim, Daniel Büche, René M. Rossi, Christina M. Spengler

**Affiliations:** 1 Empa, Swiss Federal Laboratories for Materials Science and Technology, Laboratory for Biomimetic Membranes and Textiles, St. Gallen, Switzerland; 2 Exercise Physiology Lab, Institute of Human Movement Sciences and Sport, ETH Zurich, Zurich, Switzerland; 3 Kliniken Valens, St. Gallen, Switzerland; 4 Zurich Center for Integrative Human Physiology (ZIHP), University of Zurich, Zurich, Switzerland

**Keywords:** autonomic nervous system, ECG, fatigue, HRV, longitudinal study, physiological variables, post-COVID-19, wearables

## Abstract

**Introduction:**

Psychophysiological research increasingly recognizes the multidimensional nature of subjective experiences such as fatigue and their relevance for autonomic regulation. However, little is known about how different dimensions of fatigue are associated with changes in core cardio-respiratory variables in clinical populations. This longitudinal study aimed to investigate the associations between three dimensions of fatigue – physical, cognitive, and emotional – and core physiological variables in post-COVID-19 patients. Physiological variables were collected during both daytime and nighttime periods to examine the individual and combined effects of fatigue dimensions.

**Methods:**

To this end, thirty post-COVID-19 patients (male: 11, female: 19, age: 44.1 ± 12.7 years) participated in the study during their 29.6 ± 10.2 days stay in a rehabilitation clinic. Four times a day, they reported their fatigue level using a mobile app. Physiological variables, i.e., electrocardiogram (ECG), respiratory rate, and blood oxygen saturation were recorded continuously via wearable sensors during at least 2 monitoring weeks that were 2 weeks apart. Linear mixed models were used to assess associations between daily averages of each fatigue dimension and physiological variables, adjusted for potential confounders.

**Results:**

The interaction of multiple fatigue dimensions demonstrated more consistent associations with physiological changes than single dimensions independently. Daily changes in fatigue were associated with fluctuations in physiological variables, particularly during sleep. Specifically, daily changes in resting heart rate (*P* < .01), sample entropy of RR intervals (*P* = .04), and LF peak frequency (*P* = .047), as well as overnight changes in LF/HF ratio (*P* = .046) and sample entropy of RR intervals (*P* < .001), showed significant linear associations with changes in the interaction among fatigue dimensions. Patterns suggest a shift toward sympathetic dominance with higher physical fatigue, and a shift toward parasympathetic dominance with increases in both cognitive and emotional fatigue. Additional trends (adjusted *P* < .10) supported the interpretation that interaction effects between fatigue components play a critical role in autonomic dynamics.

**Discussion:**

This study highlights the importance of investigating fatigue as a multidimensional construct in this patient group, enhancing understanding of its relationship with autonomic regulation and supporting more personalized approaches to monitoring and managing fatigue in post-COVID-19 patients.

## 1 Introduction

Fatigue is a multidimensional symptom commonly associated with a wide range of physical and psychological conditions. It is one of the most frequent and debilitating symptoms reported by individuals with post-COVID-19 syndrome, significantly impairing daily functioning and quality of life (1–3). Despite its clinical relevance, fatigue remains difficult to quantify objectively due to its inherently subjective nature and complex interactions with psycho-physiological changes. Recent advances in wearable technology have enabled continuous, unobtrusive monitoring of core physiological variables in real-world settings, offering new opportunities to investigate how fatigue is reflected in underlying physiological processes (4).

Understanding these physiological correlates may prove critical for the detection and quantification of fatigue, particularly in post-COVID-19 and other chronically ill populations. Among the most studied physiological markers of post-COVID-19 fatigue are heart rate variability (HRV) parameters, as autonomic dysregulation – including reduced parasympathetic and heightened sympathetic activity – has been frequently reported in post-COVID-19 patients (5, 6).

Previous research has largely relied on case-control designs conducted under controlled conditions, such as during clinical assessments or standardized tests. These studies have identified reduced HRV in post-COVID-19 patients compared to controls, including lower values in certain time domain variables of HRV, i.e., the square root of the mean squared differences between successive RR intervals (RMSSD), the standard deviation of RR intervals (SDNN), and the proportion of successive RR intervals pairs differing more than 50 ms (pNN50), specifically the power within the very low, low, and high frequency bands (7–9). However, most of this evidence is based on short-duration electrocardiogram (ECG) recordings, typically lasting less than 24 h. Further investigation is needed to determine whether such autonomic differences are also observed between fatigued and non-fatigued patients (10, 11). Moreover, studies examining how physiological variables vary within individuals across different levels of fatigue are still lacking.

The present study aims to address this gap by investigating the associations between daily self-reported fatigue in post-COVID-19 patients – across its cognitive, emotional, and physical dimensions – and continuously monitored core physiological variables collected during both day and night over at last 2 weeks. The findings of this study may offer initial evidence identifying the physiological variables that best reflect changes in fatigue, which could be valuable for developing tools for personalized fatigue monitoring and management in patient populations.

## 2 Methods

### 2.1 Study design

This study utilized data collected during the clinical trial conducted between March 2023 and July 2024 at Klinik Gais, a rehabilitation clinic located in Gais, Switzerland. The trial included 30 patients with post-COVID-19 fatigue, defined as a score of 5 or higher on either the Single-Item Fatigue (SIF) or the Fatigue Severity Scale upon admission. Exclusion criteria included the existence of comorbidities that could significantly affect physiological variables, with pre-existing health conditions in which fatigue is a primary symptom as outlined in the EUROMENE guidelines (12). In addition, pregnant patients and patients who were unable to use a mobile phone or to wear or handle the monitoring systems were excluded.

Patients reported their levels of fatigue four times daily (before breakfast, lunch, dinner, and bedtime) via a mobile application installed on their personal smartphones (m-path, KU Leuven, Leuven, Belgium), throughout their entire stay at the clinic.

**Figure 1:**
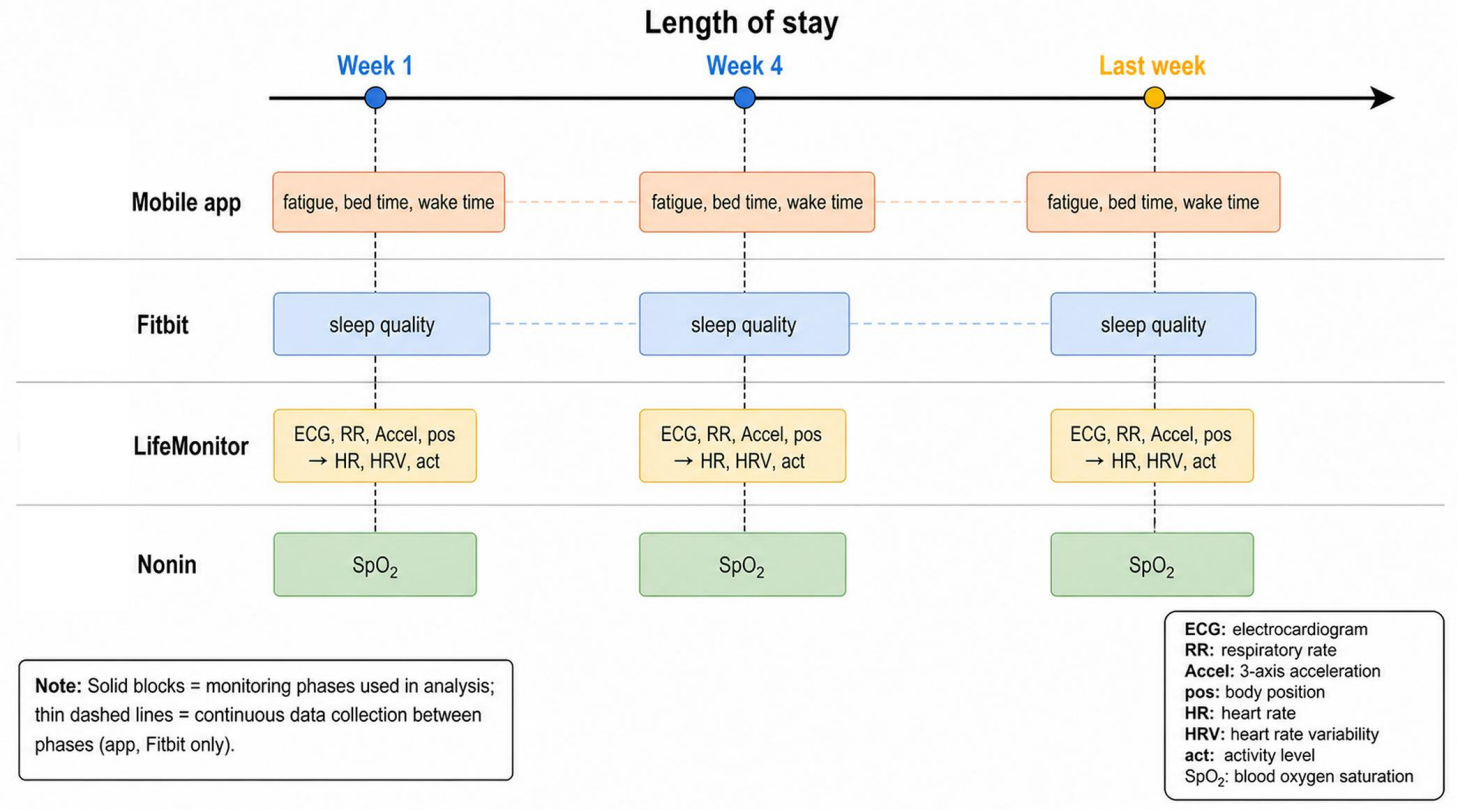
Study design: monitoring weeks, devices, and monitored physiological variables. Life Monitor: eqO2+ LifeMonitor; Nonin: Nonin Wristox2 3150; Fitbit: Fitbit Charge 5.

Self-reported fatigue was complemented by continuous monitoring of core physiological variables. To reduce participant burden, patients were monitored for one week every three weeks during their rehabilitation stay (e.g., weeks 1, 4, and the final week, depending on individual stay duration; hereinafter referred to as monitoring weeks), as shown in Figure 1.

During monitoring weeks, patients were asked to wear a monitoring device for 24 h on seven consecutive days, except when bathing. The device recorded electrocardiogram (ECG), respiratory rate, motion, and body position (eqO2+ LifeMonitor, Equivital Inc, New York, NY, USA). Validation studies demonstrated that the eqO2+ LifeMonitor RR interval validity error was not significantly different from zero and remained within clinically acceptable limits, varying according to artifact content (13–15). Nocturnal blood oxygen saturation was recorded via a wrist-worn, medical pulse oximeter (Nonin WristOx2 3150, Nonin Medical Inc, Plymouth, MN, USA). It has an accuracy of ±2% according to the manufacturer and has been shown to be suitable for monitoring in unattended settings (16). In addition, patients wore a smartwatch (Fitbit Charge 5, Fitbit Inc., San Francisco, CA, USA) during their entire stay. This device provided a sleep quality evaluation based on recordings of heart rate and activity The validity of the sleep evaluation has been previously investigated and Fitbit Charge 5 was recognized as a valid tool in research for the monitoring of sleep stages (17–19).

### 2.2 Fatigue questionnaires

The physical, cognitive, and emotional dimensions of fatigue were assessed using corresponding items from the SIF (20), presented as visual analogue scales (0 = no fatigue; 10 = strong fatigue). The items were: “I feel fatigued because I have ‘no strength’, because my body is weak, because my muscles are weak” for physical fatigue, “I feel fatigued because I am fatigued ‘in my head’, because I have trouble concentrating, because my comprehension is slowed down” for cognitive fatigue, and “I feel fatigued because I feel ‘no joy’, because I have no pleasure, because I have no drive, because nothing makes sense (anymore)” for emotional fatigue. All questions were administered in German.

### 2.3 Physiological and motion data recording and analysis

To estimate daily values of the physiological variables, medians were computed separately for wake and sleep periods using raw data. For daytime values, only data collected while patients were stationary, i.e., not walking or otherwise moving around for more than five consecutive minutes were included. For nighttime values, data points recorded while patients were detected to be upright were excluded, thereby omitting periods of nocturnal activity such as toilet visits. Sleep and wake periods were defined based on self-reported bedtimes and wake times, which patients entered each morning via the mobile app.

#### 2.3.1 Heart rate and HRV

Heart rate and HRV parameters were computed using Kubios HRV Scientific (Kubios Oy, Kuopio, Finland), based on ECG raw signals recorded from a single lead of eqO2+ LifeMonitor at a sampling frequency of 256 Hz. The software analyzed RR intervals on a beat-by-beat basis, using 5-minute signal segments to assess HRV, without artifact correction.

The time-domain parameters included the mean heart rate, RMSSD, SDNN, pNN50, and deceleration capacity (DC)/ acceleration capacity [AC (21, 22),], which measure the lengthening/shortening of RR intervals within 4 successive beats. AC and DC provide insight into how effectively the heart can increase or decrease its rate in response to varying physiological demands.

Frequency-domain parameters included the peak frequency on the low frequency (LF, 0.04–0.15 Hz) and high frequency (HF, 0.15–0.4 Hz) bands, LF and HF normalized power, and LF/HF ratio.

Nonlinear parameters Poincaré plot short-term variability (SD1), Poincaré plot long-term variability (SD2), SD1/SD2 ratio, as well as approximate and sample entropy, both measures the complexity or irregularity of the signal, were also considered.

#### 2.3.2 Respiratory rate

Respiratory rate (fR) was derived from changes in the thoracic circumference detected by the expansion sensor (resistive strain gauge) on the eqO2+ LifeMonitor. For analysis, respiratory rate was calculated using a 60-second moving average, updated every 5 s. Variability measures of fR – including standard deviation, coefficient of variation, and sample entropy – were computed separately for daytime and nighttime periods and added to the analysis.

#### 2.3.3 Overnight blood oxygen saturation

Oxygen saturation was recorded at a sampling frequency of 1 Hz. Artifacts were automatically detected by the Nonin device and excluded from the analysis.

#### 2.3.4 Body position and motion

Body position and motion data were obtained from eqO2+ LifeMonitor, which includes a 3-axis accelerometer with 0.2 Hz sampling frequency to compute posture and motion providing 5-second averages. Activity level was quantified as the median inertial acceleration, calculated from 3-axis accelerometer data recorded by the eqO2+ LifeMonitor, after applying third-order elliptical filter (23). The physiological variables included in the analysis are listed in Figure 1.

### 2.4 Statistical analysis

Linear mixed models were employed to investigate the association between daily self-reported fatigue – calculated as the average of all four reports on a given day – and daily medians of the physiological variables. The different dimensions of self-reported fatigue, along with their interactions, were modelled as independent variables, while the physiological variables served as dependent variables, as described in the equation below:

$$Y_{ij}\;=\;\beta_{0}\;+\;\beta_{1}Covariates_{ij\;}\;+\;\beta_{2}(Physical\;\times \;Cognitive\;\times \;Emotional{)}_{ij\;}\;+\;u_{0i}\;+\;\epsilon_{ij}$$

In this model, $Y_{ij}$ represents the physiological variable for subject i on day j. $\beta_{0}$ represents the fixed intercept, $\beta_{1}$ denotes the fixed effects of covariates, and $\beta_{2}$ captures the fixed effect of the three-way interaction between physical, cognitive, and emotional fatigue on the outcome variable $Y_{ij}$. Covariates are included as fixed effects. The term $(Physical\;\times \;Cognitive\;\times \;Emotional{)}_{ij\;}\;$ represents the interaction between physical, cognitive, and emotional fatigue, capturing their joint effect on the outcome. $u_{0i}$ is a random intercept accounting for subject-specific variability, and $\epsilon_{ij}$ is the residual error term. For each physiological variable, a mixed-effects model with a random intercept and fixed slope was fitted. This approach allowed us to assess the overall association between fatigue and physiological variables across patients, while accounting for individual differences in baseline physiological levels.

Models included age, sex (coded as 1 for male, 0 for females), body mass index, baseline functional capacity (assessed via the 6-minute walking distance), use of beta-blockers (coded as 1 for users, 0 for non-users), use of antidepressants (coded as 1 for users, 0 for non-users), time (number of days since study start), time awake prior to bedtime, sleep duration, sleep quality, and the number of fatigue self-reports recorded per day as covariates to account for their potential effects on the physiological variables. Daily fatigue levels were modelled using either daytime data from the same day or nighttime data from the preceding sleep period. Accordingly, models based on daytime data were additionally adjusted for the activity level on that day, while models based on nighttime data were adjusted for activity levels from the previous day.

Data was standardized using z-score normalization prior to model fitting. Model assumptions were evaluated through residual diagnostics. Logarithmic (for RMSSD, SDNN, DC, LF/HF ratio, SD1, SD2, fR, fR coefficient of variation, and fR sample entropy) or log-plus-one (for pNN50, and AC) transformations were applied to the dependent variable, when necessary, to improve the distribution of model residuals. Confidence intervals for fixed-effect estimates were obtained using parametric bootstrap resampling with 1,000 iterations, providing a robust approach given the small sample size and the presence of skewed data (24, 25). Two-sided p-values were derived empirically from the bootstrap distributions (26) and subsequently adjusted for multiple comparisons using the Benjamini–Hochberg false discovery rate procedure (27).

Due to the clinical heterogeneity of the post-COVID-19, the influence of individual patients on model estimates was evaluated by computing Cook's distance at the subject level (28, 29). A threshold of 0.5 was used to identify potentially influential cases, based on both standard guidelines (30) and visual inspection of the distribution of Cook's distances in the dataset. This procedure was performed to ensure that atypical cases did not disproportionately bias the results and to assess the robustness of the findings to potentially influential observations.

All data preprocessing procedures were performed using MATLAB R2023b (The MathWorks, Inc., Natick, MA, USA), while statistical analyses were performed in R (version 4.4.1; R Core Team, 2024). Linear mixed models were fitted using the lme4 package (31), bootstrapped confidence intervals were obtained with bootMer, and subject-level influence diagnostics were performed using the influence.ME package (29).

Results are reported based on the full dataset when the exclusion of influential data points did not alter the conclusions. In cases where notable changes were observed, results from the cleaned dataset are reported. Model estimates (*β*) are reported with corresponding 95% confidence intervals (95% CI). For log-transformed variables, percent changes were derived by back-transforming the model estimates. For variables not log-transformed, percent changes were calculated as the ratio of the estimate to the model intercept. Statistical significance was defined as a two-sided p-value equal or lower than 0.05. Unless otherwise specified, data are presented as mean ± standard deviation (SD) when normally distributed or the median (interquartile range, IQR) otherwise.

## 3 Results

### 3.1 Dataset description

Patients’ characteristics and dataset overview are presented in Table 1. After data processing, data from the 30 patients yielded 283 monitored days and 259 monitored nights. On average, each patient contributed with 9.4 daytime and 8.6 nighttime observations. Overall, patients reported high levels of physical (6.4 ± 1.7 in the daytime dataset, 6.4 ± 1.6 in the nighttime dataset) and cognitive fatigue (5.0 ± 2.2), and lower levels of emotional fatigue (1.1 (0.0–3.8)).

**Table 1:** Patient demographics and dataset description. Associations between daily fatigue levels and physiological variables were investigated using either daytime variables from the same day or nighttime variables from the preceding sleep period.

Baseline characteristics of patients	Data
Number of patients (male/female)	30 (11/19)
Age (years)	44.1 ± 12.7
BMI (kg/m^2^)	26.2 ± 5.7
Study duration (days)	29.6 ± 10.2
6-minute Walking Distance (m)	485.1 ± 111.0
Beta-blocker use, n	4 (13%)
Antidepressant use, n	10 (33%)

**Measures during the monitoring period**	**Daytime dataset**	**Daytime dataset**
Total number of monitored days	283	259
Monitored days per patient, mean (range)	9.4 (3–18)	8.6 (4–16)
Time awake prior to bedtime (h)	15.7 ± 1.4	15.7 ± 1.4
Sleep duration (h)^a^	8.3 ± 1.4	8.3 ± 1.4
Sleep quality (0-59 = Poor, 60–79 = Fair, 80–89 = Good, 90–100 = Excellent)^a^	93.1 ± 2.6	92.9 ± 2.7
Activity (mG)^b^	9.4 ± 2.3	9.4 ± 2.6
Number of fatigue self-reports per day	4 (4–4)	4 (4–4)
Physical Fatigue (0 = no fatigue, 10 = strong fatigue)	6.4 ± 1.7	6.4 ± 1.6
Cognitive Fatigue (0 = no fatigue, 10 = strong fatigue)	5.0 ± 2.2	5.0 ± 2.2
Emotional Fatigue (0 = no fatigue, 10 = strong fatigue)	1.1 (0.0–3.8)	1.1 (0.0–3.8)

BMI, body mass index.

a Sleep duration and sleep data quality refer to the preceding sleep period for the daytime dataset and to the same night for the nighttime dataset.

b Activity refers to the same day for the daytime dataset and to the previous day for the nighttime dataset. Unless otherwise stated, data are presented as mean ± standard deviation (SD) when normally distributed, or as median (interquartile range, IQR) when not.

One patient exerted extraordinary influence on the results, as evidenced by a reversal in the direction of statistically significant associations when this patient was excluded from the analysis. Specifically, the reversal was observed between self-reported fatigue and nighttime DC, LF and HF normalized power, and LF/HF ratio. To ensure the robustness of the findings, this influential patient was excluded from all HRV parameter models using nighttime data. The estimated average value of each physiological variable, when all predictors are at their reference level (i.e., zero after standardization), is presented in Table 2.

**Table 2:** Linear mixed model intercept estimates for each physiological variable across the two datasets (Daytime and Nighttime).

Parameter	Daytime Intercept [95% CI]	Nighttime Intercept [95% CI]
SpO_2_ (%)	NA	93.37 [93.08, 93.56]
HR (bpm)	78.63 [76.86, 80.51]	63.38 [60.98, 65.40]
RMSSD (ms)	30.69 [27.53, 34.10]	37.43 [34.75, 41.19]
SDNN (ms)	36.22 [33.66, 39.30]	39.55 [36.73, 42.56]
pNN50 (%)	5.09 [4.34, 5.97]	8.79 [7.77, 10.25]
DC (ms)	20.55 [19.07, 22.31]	26.45 [24.70, 28.38]
AC (ms)	−21.77 [−25.46, −18.54]	−28.70 [−31.50, −25.79]
LF peak f (Hz)	0.08 [0.08, 0.08]	0.062 [0.061, 0.063]
HF peak f (Hz)	0.22 [0.21, 0.23]	0.26 [0.25, 0.26]
LF n.u. power	67.61 [65.41, 69.76]	58.41 [56.18, 59.96]
HF n.u. power	31.38 [29.69, 33.08]	41.64 [40.39, 43.31]
LF/HF ratio	2.22 [2.02, 2.42]	1.42 [1.30, 1.51]
SD1 (ms)	21.73 [19.66, 24.32]	26.51 [24.40, 29.13]
SD2 (ms)	45.31 [42.18, 48.76]	47.97 [44.88, 51.07]
SD2/SD1 ratio	2.04 [1.96, 2.14]	1.73 [1.66, 1.78]
ApEn	1.11 [1.09, 1.13]	1.08 [1.07, 1.09]
SampEn HR	1.41 [1.37, 1.46]	1.55 [1.52, 1.60]
fR (brpm)	14.02 [13.52, 14.51]	9.55 [8.89, 11.00]
std fR (brpm)	5.28 [4.98, 5.52]	5.63 [5.33, 5.91]
CV fR	0.38 [0.35, 0.41]	0.56 [0.50, 0.61]
SampEn fR	0.05 [0.04, 0.06]	0.03 [0.02, 0.03]

NA, not applicable; SpO2, blood oxygen saturation; fR, respiratory rate; HR, heart rate; DC, deceleration capacity of heart rate; AC, acceleration capacity of heart rate; LF peak f, low frequency band peak frequency; HF peak f, high frequency band peak frequency; LF n.u. power, low frequency normalized power; HF n.u. power, high frequency normalized power; ApEn, approximate entropy; SampEn, sample entropy; std, standard deviation; CV, coefficient of variation.

### 3.2 Associations between fatigue and physiological variables

The results for the interaction terms between physical, cognitive, and emotional fatigue, and their associations with the physiological variables, are shown in Table 3. Only associations with confidence intervals excluding zero are presented, with complete results provided in Supplementary Figures S1, S2.

**Table 3:** Estimated effects and 95% confidence intervals from models including physical, cognitive, and emotional fatigue, and their interactions. Only associations with confidence intervals that do not include zero are shown. Estimates represent the percent change per one standard deviation increase in the scaled predictors. × represents the interaction between the different fatigue dimensions, with ‘Three-way interaction’ referring to the interaction between the three dimensions of fatigue (Physical×Cognitive×Emotional). .

Dataset	Fatigue Dimension (independent predictor)	Parameter	Estimate [95% CI]	*P*
Daytime	Emotional	LF peak f	3.69 [0.12, 6.03]	.15
		HF peak f	4.31 [0.03, 8.51]	.15
	Cognitive	fR	3.69 [0.17, 6.53]	.12
	Cognitive × Emotional	HR	−4.17 [−6.93, −1.46]	<.01^b^
		SampEn HR	4.46 [1.17, 8.78]	.04^a^
	Physical × Emotional	LF peak f	−3.52 [−5.30, −0.55]	.047^a^
		CV fR	−10.04 [−15.97, −0.05]	.15
	Physical × Cognitive	HR	2.11 [0.20, 4.43]	.08
	Three-way interaction	HR	3.03 [1.73, 5.36]	<.001^c^
		LF peak f	1.67 [0.04, 2.98]	.15
Nighttime	Emotional	LF n.u. power	−4.29 [−11.26, −1.05]	.07
		HF n.u. power	4.96 [1.38, 11.94]	.08
		LF/HF ratio	−9.84 [−22.36, −2.22]	.046^a^
	Cognitive × Emotional	AC	−0.40 [−0.70, −0.01]	.13
	Physical × Emotional	SDNN	−8.56 [−16.47, −0.79]	.10
		DC	−8.23 [−15.88, −1.44]	.06
		SD2	−8.39 [−15.81, −1.47]	.06
	Physical × Cognitive	SpO_2_	−0.23 [−0.45, −0.04]	.06
		SDNN	7.07 [0.95, 17.52]	.08
		LF peak f	2.41 [0.49, 4.83]	.07
		SD1	7.80 [0.04, 19.94]	.14
		SD2	7.01 [1.66, 15.92]	.05
		SampEn HR	−4.96 [−7.28, −2.54]	<.001^c^
	Three-way interaction	pNN50	−15.75 [−26.62, −1.31]	.11
		DC	−8.79 [−15.14, −1.21]	.08
		AC	0.39 [0.04, 0.71]	.09

SpO2, blood oxygen saturation; fR, respiratory rate; HR, heart rate; AC, acceleration capacity of heart rate; DC, deceleration capacity of heart rate; LF peak f, low frequency band peak frequency; HF peak f, high frequency band peak frequency; LF n.u. power, low frequency normalized power; HF n.u. power, high frequency normalized power; SampEn HR, sample entropy; CV, coefficient of variation.

While only one significant effect was observed for the single dimensions of fatigue, several joint effects emerged from the interaction between fatigue dimensions. Fatigue was found to be significantly associated with changes in heart rate during daytime. Specifically, a joint increase in cognitive and emotional fatigue was associated with a 4% decrease in heart rate (*β* = −3.28 bpm, 95% CI: −5.44 to −1.15, *P* < .01) and a 4% increase in heart rate sample entropy (*β* = 0.06, 95% CI: 0.01 to 0.12, *P* = .04). In contrast, the three-way interaction between physical, cognitive, and emotional fatigue was associated with a 3% increase in heart rate (*β* = 2.38 bpm, 95% CI: 1.36 to 4.21, *P* < .001). Additionally, model estimates revealed a significant decrease in LF peak frequency with the joint increase in physical and emotional fatigue (*β* = −0.003 Hz, 95% CI: −0.004 to −0.0004, *P* = .047), and suggested an increase in heart rate with the joint increase in physical and cognitive fatigue (*P* = .08).

Potential associations were identified from confidence intervals that indicated a directed change in the physiological variables with changes in the fatigue dimensions, specifically between LF/HF peak frequency and emotional fatigue; fR and cognitive fatigue; fR coefficient of variation and the joint effect of physical and emotional fatigue; as well as LF peak frequency and the three-way interaction between physical, cognitive, and emotional fatigue, as they did not include zero. However, after correction for multiple comparisons, the adjusted p-values for these associations exceeded 0.10 (see Table 3).

More associations between physiological variables and fatigue were observed during the nighttime period. Increased emotional fatigue was associated with decreased LF/HF ratio (*β* = −0.10, log-transformed, 95% CI: −0.25 to −0.02, *P* = .046). It was also associated with trends toward decreased LF normalized power (*P* = .07) along with increased HF normalized power (*P* = .08). A joint increase in physical and cognitive fatigue was associated with lower heart rate sample entropy (*β* = −0.08, 95% CI: −0.11 to −0.04, *P* < .001) during sleep. This interaction also showed trends toward decreased blood oxygen saturation (*P* = .06), as well as increased SDNN (*P* = .08), LF peak frequency (*P* = .07), and SD2 (*P* = .05).

Besides, the joint increase in physical and emotional fatigue was associated with a trend toward decreased SDNN (*P* = .10), DC (*P* = .06), and SD2 (*P* = .06); and the three-way interaction between physical, cognitive, and emotional fatigue with lower DC (*P* = .08), alongside increases in AC (*P* = .09).

## 4 Discussion

In this study, we investigated the associations between the level of fatigue (physical, cognitive, and emotional) reported throughout the day and core cardio-respiratory variables in a cohort of post-COVID-19 patients. Changes in daily fatigue were significantly associated with changes in daytime heart rate, heart rate sample entropy, and LF peak frequency, as well as with reductions in nighttime LF/HF ratio and heart rate sample entropy. Our findings suggest that, while only one significant association was observed between individual dimensions of fatigue and the physiological variables, the joint variation in fatigue dimensions is mirrored by measurable physiological alterations, particularly during sleep.

Daytime data showed that a joint increase in cognitive and emotional fatigue was associated with a decreased heart rate (*P* < .01), whereas the three-way interaction between physical, cognitive, and emotional fatigue was linked to increased heart rate (*P* < .001). This suggests that physical fatigue moderates the effect of cognitive and emotional fatigue on heart rate, attenuating the reduction observed when physical fatigue is not elevated. Increased resting heart rate is an indicator of increased sympathetic drive (32, 33). Thus, these findings suggest that elevated physical fatigue moderates the parasympathetic activation associated with the combined effect of cognitive fatigue and emotional fatigue, attenuating this response and contributing to a shift toward sympathetic dominance.

The decrease in heart rate caused by the combined effect of cognitive and emotional fatigue was accompanied by an increased heart rate sample entropy (*P* = .04). Since sample entropy reflects the complexity or irregularity of RR intervals (34, 35), this pattern – lower heart rate and higher sample entropy – may indicate enhanced parasympathetic activity and irregularity in heart rate dynamics. The observed decrease in LF peak frequency in response to the combined effect of physical and emotional fatigue may also indicate reduced sympathetic activity and a concomitant parasympathetic activation (36–38).

The analysis of the nighttime data revealed a significant reduction in LF/HF ratio associated with higher emotional fatigue levels (*P* = .046) alongside a decrease in LF normalized power, and an increase in HF normalized power, suggesting a shift toward parasympathetic modulation (36, 39). The observed increase in parasympathetic activity during sleep associated with higher emotional fatigue in this population represents a distinct pattern that contrasts with classic chronic fatigue syndrome, in which reduced nocturnal parasympathetic activity is typically reported (40, 41). This divergence may reflect a compensatory mechanism – an attempt by the body to restore autonomic balance and promote recovery in response to the autonomic dysfunction characteristic of post-COVID-19 syndrome.

A joint increase in physical and cognitive fatigue was associated with decreased heart rate sample entropy (*P* < .001). Confidence intervals suggest a potential trend toward increased overall (SDNN) and long-term HRV (SD2), as well as increased LF peak frequency and reduced blood oxygen saturation. While the increased HRV during sleep suggests an increased parasympathetic drive, linked to restorative sleep (41, 42), higher LF peak frequency and reduced entropy during sleep may reflect increased sympathetic activity (43–46), possibly compromising the restorative benefits that would normally alleviate fatigue (47–49). Decreased blood oxygen saturation has also been linked to increased fatigue (50–53).

Additional trends observed in the nighttime data – such as lower overall (SDNN) and long-term HRV (SD2), decreased DC, and elevated AC in response to the joint increase in physical and emotional fatigue, as well as the three-way interaction between physical, cognitive, and emotional fatigue – are consistent with a shift toward sympathetic drive and reduced parasympathetic modulation (19, 34, 37).

Altogether, our findings indicate that an elevated level of self-reported fatigue across its different dimensions is associated with altered autonomic regulation during both wakefulness and sleep. Specifically, an elevated level of physical fatigue appears to be linked to a shift toward sympathetic drive, while an elevated level of cognitive and emotional fatigue is more closely associated with parasympathetic modulation. However, these findings warrant further investigation.

Several factors may limit the generalizability of our findings. First, the study was performed in a specific cohort of patients, suffering from a chronically elevated level of fatigue. Second, although physiological variables were continuously collected and analyzed using robust modelling approaches, several factors could have influenced model estimates. These include improper sensor placement or unaccounted confounders, such as daily medication intake, therapy plans or hormonal-status-related physiological variability among female participants due to the inclusion of participants across reproductive and menopausal stages. Additionally, fR variability was not derived from breath-by-breath measurements, unlike heart rate variability, which may have limited the precision of these estimates. Furthermore, some associations identified were trends rather than statistically significant findings after multiple testing correction and should therefore be interpreted with caution. One extraordinary influential subject was identified whose inclusion reversed significant nighttime associations; while this case was excluded from nighttime HRV-models to ensure robustness, its influence underscores the extent of individual variability in post-COVID-19 fatigue. Lastly, generalizability may also be limited by the monitoring setting.

The present findings align with previous research showing altered autonomic function in individuals with post-COVID-19 fatigue (10, 31). However, in contrast to previous findings, fatigue in the present study was associated not only with reduced parasympathetic and increased sympathetic activity, but also with the opposite pattern, depending on the specific fatigue dimension. Given that fatigue in post-COVID-19 may share underlying characteristics with fatigue observed in other conditions while also presenting condition-specific features, further investigation is needed to determine whether the patterns identified in this study are generalizable across different patient populations. Ongoing and future long-term wearable-based studies involving both post-COVID-19 and other clinical populations may help clarify whether these findings reflect broader fatigue-related mechanisms or characteristics specific to post-COVID-19 (54, 55).

While most prior studies used short-duration ECG recordings under well-controlled conditions, our study extends this evidence by demonstrating associations between day-to-day fluctuations in self-reported fatigue and core physiological variables captured through long-term monitoring in a semi-controlled clinical environment. Notably, we found distinct physiological signatures linked to different fatigue dimensions, offering a more nuanced perspective on how fatigue manifests physiologically.

## 5 Conclusion

This study provides evidence that the combined effects of multiple dimensions of self-reported fatigue are reflected in patients’ physiological variables, particularly during nighttime. Effects of individual fatigue dimensions, as well as their interactions, were linked to alterations in autonomic indicators such as heart rate, heart rate sample entropy, LF peak frequency, and LF/HF ratio. These findings underscore the importance of considering the multidimensional and interacting aspects of fatigue when investigating its physiological effects in post-COVID-19 and others clinical populations. Future research should aim to replicate these findings in larger cohorts, using longitudinal designs to clarify temporal dynamics and potential causal relationships, while also incorporating a broader range of physiological variables (e.g., blood pressure, motion) to deepen the understanding of underlying mechanisms.

## Data Availability

The raw data supporting the conclusions of this article will be made available by the authors, without undue reservation.
