# Supplementary material for: Associations between multidimensional fatigue and wearable-derived cardio-respiratory variables in post-COVID-19 patients: an observational study
Source: Front Digit Health. 2026 Jul 16;8:1804993. doi: 10.3389/fdgth.2026.1804993 (PMC13422486; doi:10.3389/fdgth.2026.1804993)
Supplement: Supplementary file 1 [file Datasheet1.docx]

Supplementary Material


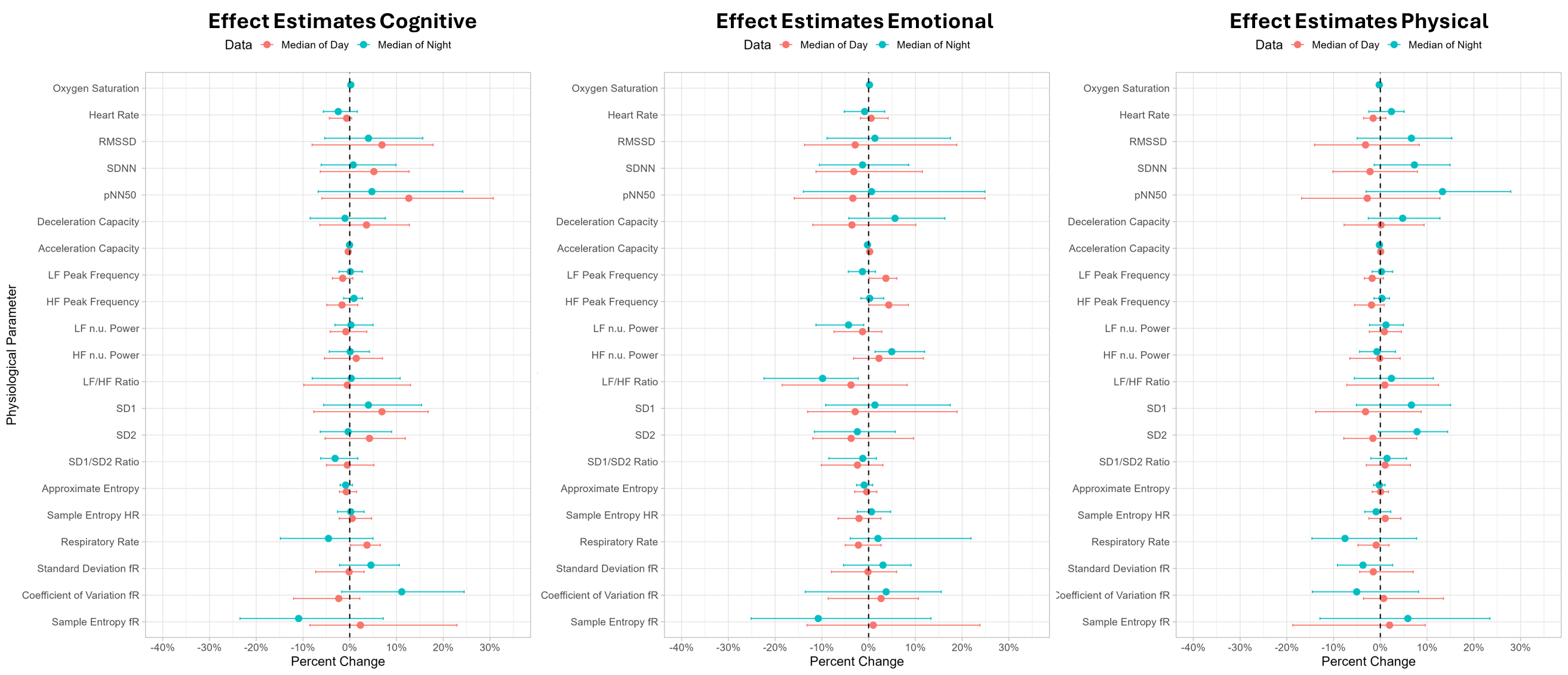


**Supplementary Figure 1**. Estimated effects (percent change) and confidence intervals by model for cognitive, emotional, and physical fatigue. LF n.u. power: low frequency normalized power; HF n.u. power: high frequency normalized power; HR: heart rate; fR: respiratory rate.


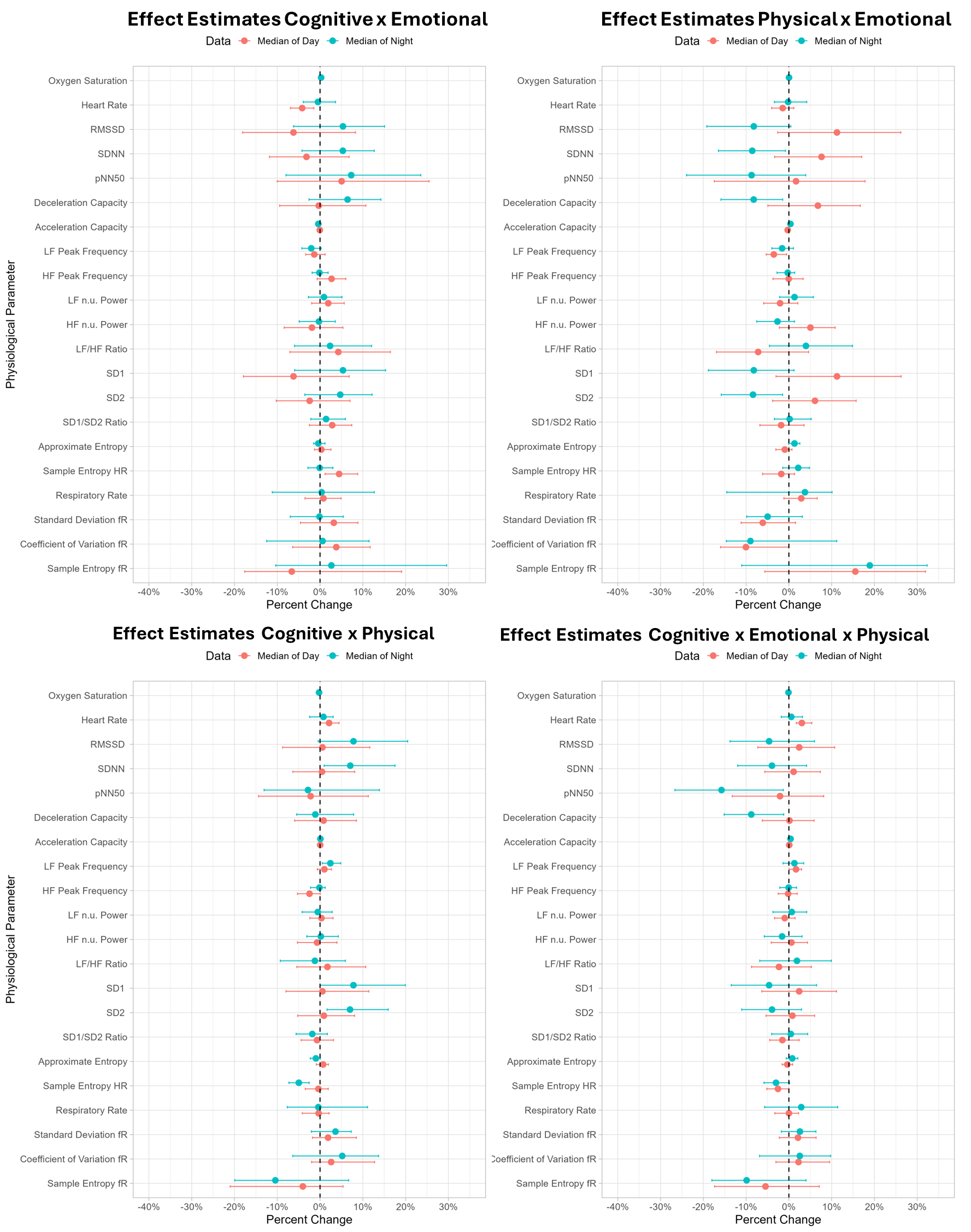


**Supplementary Figure 2**. Estimated effects (percent change) and confidence intervals by model for interactions between cognitive, emotional, and physical fatigue. LF n.u. power: low frequency normalized power; HF n.u. power: high frequency normalized power; HR: heart rate; fR: respiratory rate.
